# Methodology of estimation of temperature mode in the 2xBgu type railway braking system

**DOI:** 10.1038/s41598-022-25283-2

**Published:** 2022-12-02

**Authors:** Aleksander Yevtushenko, Michal Kuciej, Piotr Grzes, Piotr Wasilewski

**Affiliations:** 1 Faculty of Mechanical Engineering, Bialystok University of Technology (BUT), 45C Wiejska Street, 15-351 Bialystok, Poland; 2 Dellner Frimatrail Frenoplast S.A., 15 Watykańska Street, 05-200 Majdan, Poland

**Keywords:** Mechanical engineering, Engineering, Materials science

## Abstract

The article presents finite element models of the 2xBgu type tread brake for the simulation of extended repeated frictional heating carried out on a full-scale inertia dynamometer. The numerical calculations were conducted for the brake blocks made of two organic composite materials newly developed specifically for this study. The transient temperature changes obtained from the 2D axisymmetric and 3D finite element analyses and experimental data agreed well during continuous process of about 1200 s. Simulation of such a long period of braking sequence required introducing simplifications in the boundary conditions in the contact area, convection cooling, arrangement of the model (2D axisymmetric, 3D). The focus was laid on representation of variation of the coefficient of friction and the temperature dependence of the properties of the friction materials during braking. The carried out research indicates limitations in the finite element analysis and directions of necessary improvements in modelling as well as measurements with the use of embedded thermocouples.

## Introduction

In the European Union, freight trains are mainly equipped with block braking systems. In 75% of wagons cast iron is used as the friction material, which generates significant noise during braking^1,2^. Regulations in the European Union concerning the reduction of noise levels^3,4^ are forcing the accelerated replacement of cast iron brake blocks with organic composite brake blocks. That change of the friction material type, and thus also the tribological characteristics of the friction interface, created a field for further research^5–7^. The experimental results on a reduced and full-scale inertia dynamometer, for organic composite brake blocks were compared in the articles^8,9^. The studies focused on examining the influence of the selected components on the properties of the composite. It was found that the formulation of the friction material have significant influence on the temperature distribution, coefficient of friction and wear of the friction pair elements.

One of the main factors influencing the service life of the braking system is the temperature that is generated during braking^10–12^. The article by Wasilewski^13^ contains the review of the braking systems used in railway vehicles. In the introduction of the article basic information on construction of the brakes used in railway vehicles, development of friction materials, main aspects of braking performance and the design process were described. In the main part of the paper, new findings of temperature modelling in railway braking systems divided into two sections were reported. First dealt with the temperature calculations in railway tread brake while the second was concerned with corresponding calculations in the disc brake. In both an emphasis was laid on the approaches in defining the boundary conditions in the frictional heating zone. Günay et al.^14^, presented a broad review of research of braking systems (disc, tread, aerodynamic, pneumatic, electropneumatic, etc.) used in various types of railway vehicles. The authors analyzed scientific articles concerning the estimation of braking parameters (adhesion, braking force, coefficient of friction, braking distance, energy of vehicle, resistance forces acting on a vehicle, etc.), types of friction materials of brake system elements and finite element modelling of braking systems. The review study presented in Ref.^14^ on the analysis of scientific research and real applications is aimed mainly at institutions designing new types of brake systems, as well as friction materials used in brake systems in railway vehicles.

In recent years, many scientists have undertaken research related to the determination of the operational reliability of friction components of tread brakes installed on railway vehicles, and in particular the impact of the temperature generated by frictional heating during braking in a railway wheel. A three-stage approach to numerical calculation of the wheel temperature was carried out by Vakkalagadda et al.^15^. In the first stage, the friction of cast-iron brake blocks with the railway wheel and the wheel with the rail were analyzed; next, the two-dimensional boundary element method was used to determine the heat partition coefficient (HPC) between friction elements in relation to different brake block types, geometry and thermophysical properties. This allowed to determine the input parameters in the FE model (third stage) based on train running model and the boundary element method. In the third stage 4-noded linear axisymmetric quadrilateral elements were used. Apart from advanced hybrid modelling, the study provides important data on thermal behaviour by comparing configurations with one block per wheel and two opposite blocks per wheel. The results of numerical simulations and measurements for complex patterns of the velocity and braking agreed well.

The 3D FE model of the brake block for simulating the temperature changes during braking of a freight wagon was proposed by Somà^16^. The coefficient of friction and thermophysical properties of materials were constant. The duration of the applied heat flux on the friction surface of the brake block was taken from the experimental tests. The evolutions of the measured and simulated temperature coincided. Walia et al.^17^ carried out experimental measurements of temperature variations and wear of wheels and brake blocks in 1xBg configuration (one brake block per wheel) for an urban train. Additionally, a numerical model was proposed to estimate the mean temperature change on the wheel surface, which was calibrated with the experimental data.

Teimourimanesh et al.^18^, developed numerical model to calculate temperature in the brake block/wheel system in railway vehicles (metro, suburban trains) during sequential stop braking. The calculations were conducted for realistic operating parameters, considering temperature-dependent properties of materials. The paper demonstrates design directions when considering the thermal capacity of the railway wheels. The second part of the study^19^ provides examples of applications from the article^18^.

During extended and repeated braking, the treads of railway wheels are subjected to complex mechanical and thermal loads^20^. In order to prevent excessive damage to the brake system components, in particular the wheel, it is important to understand the mechanisms and operating conditions which intensify degradation of the material. Walia et al. investigated the influence of the temperature generated during braking-to-stop on the fatigue damage of the wheel tread (rolling contact fatigue) using the FE model^21^. Handa et al. in the article^22^, performed finite element analysis of temperature of the circular section of the wheel in the 1xBg braking system under constant-temperature conditions using ADINA 9.5.1 software. In the numerical model the heat input took place at the: (1) nominal contact width and (2) assuming half of the apparent contact width due to non-uniform contact pressure distribution. The brake blocks were not included in calculations and it was assumed that entire thermal energy enters the wheel. During the carried out experimental tests on a full-scale inertia dynamometer, the thermocouples were localised 10 mm under the friction surface of the wheel tread and 40 mm from the rim surface. The main goal of research was focussed on experimental assessing using a full-size railway dynamometer, the effect of the temperature of the wheel tread caused by braking on the intensity of its wear, both in contact with the brake blocks and with the rail. The role of the FEA presented in this article was to determine surface temperature due to difficulties in its measuring.

Faccoli et al. studied temperature of the wheel of the block brake by using a simplified axisymmetric model with the heat flux uniformly distributed in the circumferential direction^23^. The braking force was assumed to be constant and therefore also the deceleration was constant. In simulation only convection heat transfer was taken into account. Radiation and heat exchange to the rail (rail chill) were omitted. The heat partition during braking was constant and the fraction absorbed by brake shoes was estimated as 17% of the total heat power in the case of a new wheel and 18.6% of the total heating power in the case of a worn wheel. In the first stage, the finite element analysis of the braking-to-stop to estimate the temperature distribution in the wheel rim was performed. Next, the tests of the brake block/wheel system were carried out on a double-disc machine, where the results of the FE simulation were the input parameters^23,24^. Three different types of steel for wheels (HYPERLOS^®^—improved ER7 steel, CLASS B and SANDLOS^®^—type CLASS B steel modified) were tested with the same cast-iron brake block material. It was found, that under the influence of the phenomenon occurring during braking, the material transfer from the brake block to the wheel caused the formation of the discontinuous layer, which in turn affected the cracks nucleation in the wheel.

Mańka and Sitarz in the article^25^ developed 3D FE model of the type BA004 railway wheel without brake blocks to study temperature and von Mises stress fields. The model of the wheel was created by means of regular octahedral finite element mesh. After several preliminary numerical simulation tests the authors indicated necessity of reducing the total number of elements from 13,000 to 667 in order to enable short calculation times to be achieved. This allowed for the verification of a number of analysis variables. In calculations the heat entering the wheel (90% of total heat) did not change due to assumed constant heat partition coefficient and constant coefficient of friction. The procedure of braking was that during first stage of the analysed process 0–2700 s frictional heating took place and after that period only cooling up to 5400 s was applied. The authors described the methodology of determining thermal loads that arise during braking as well as the individual stages of creating the model based on the finite element method.

Teimourimanesh carried out comparative analysis of the temperature variations of wheel and brake blocks as a result of frictional heating during braking of a railway vehicle when using 1xBg and 2xBgu configurations^26^. In particular, the heat distribution between the brake block, wheel and rail were analyzed for the braking to stop cycle. Two thermal models were used to determine the maximum temperatures. First was the circumferential (plane) model of wheel, block and rail where the wheel treated as a thin ring and the second was an axisymmetric (axial) model of wheel, block and rail—there were no variations of temperature in the circumferential direction. It was found that the cooling effect from the rail was significant when considering the local surface temperatures, but it did not play a significant role in the estimation of the bulk temperatures generated during braking. The author pointed out also the importance of distinguishing the brake block configuration, which can strongly affect both temperature and heat distribution between the wheel and friction material. As found from the study, the 2xBg configuration reduces the temperature compared to 1xBg system.

This study is part of a series of articles devoted to finite element analyses (FEA) and experimental tests on a full-scale inertia dynamometer carried out for a railway tread brake. The main goal, starting from the article^20^, was to analyze the temperature changes in the wheel and brake blocks, calculated from FE numerical models, and the temperature measured at selected points in the wheel using embedded thermocouples. In the context of temperature changes, the input parameters of braking were assessed, i.e. the total braking force, the vehicle (sliding) velocity and the variability of the coefficient of friction. The tests were carried out for the newly developed two composite organic brake blocks manufactured for 1xBg and 2xBgu brake configurations. This work focuses on the comparative analysis of the temperature obtained from the extended (500–1200 s) experimental tests in the 2xBgu braking system and the FE models simulating the friction process based on the 2D axisymmetric and 3D spatial variants.

The novelty of the study lays in a detail representation of the time-dependent coefficient of friction for a braking lasting 1200 s, newly developed for this study friction materials with the measured temperature dependence of the thermophysical properties (thermal conductivity, specific heat and thermal diffusivity), 2xBgu configuration of the brake blocks, 3D model of the brake taking into account contact in the meaning of heat partition coefficient dependent on the varying properties of materials.

## Experimental studies of temperature distribution in a railway wheel on a full-scale inertia dynamometer

**Figure 1:**
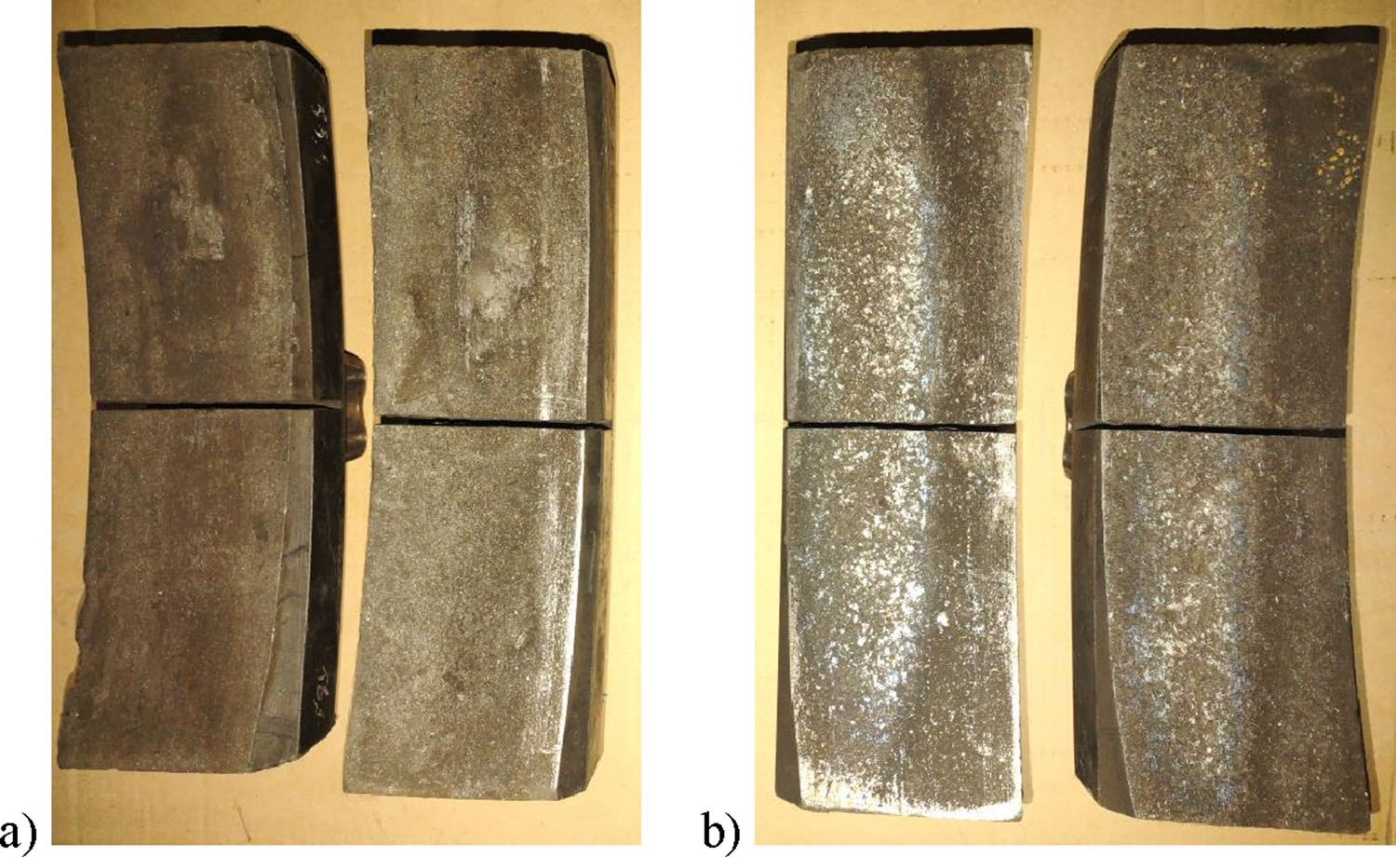
Photographs of the organic composite brake blocks after testing: (**a**) made of Material A; (**b**) made of Material B.

**Figure 2:**
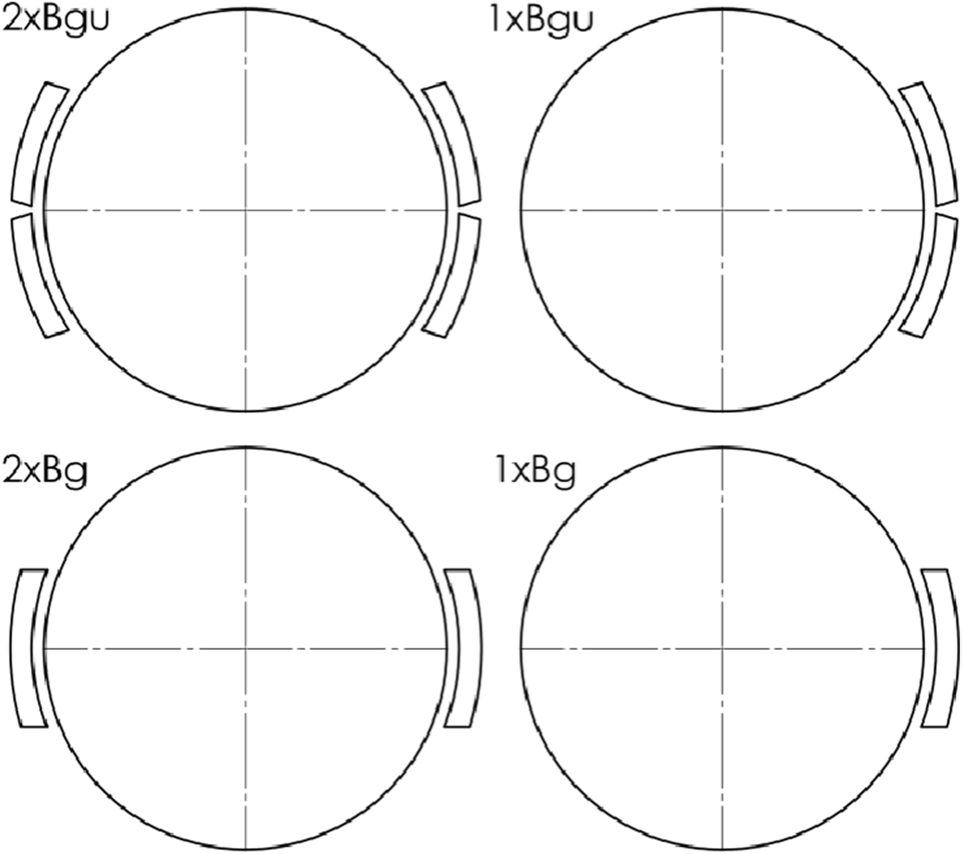
Configurations of the railway tread brake.

In order to carry out the bench tests, two sets of prototype brake blocks (Fig. 1) for the 2xBgu system (Fig. 2) were designed and manufactured in accordance with the technology used in serial production. The mixtures, from which the prototypes were made, denoted as Material A and Material B, contained reinforcing fibers (glass fiber and steel fiber, respectively) in the same mass fraction (in the range of 25–35% by weight. The precise formulation of the brake shoe material is a supplier (Dellner Frimatrail Frenoplast) trade secret and thus not disclosed in detail (see Table 1). Since steel fiber has higher density than glass fiber, its volume fraction was lower. The remaining ingredients of the formulation, their mass fraction and the production process were the same for both materials. The above friction materials have already been used in the bench tests for the 1xBg braking system^20^. The papers^9,20^ contain extended information on their material, and tribological and mechanical characteristics.

**Table 1:** The considered modification of the composition of materials^9,20^.

Ingredients	Material A (% by weight)	Material B (% by weight)
Fiberglass	25–35%	0%
Steel fiber	0%	25–35%
The other ingredients (matrix)	65–75%	65–75%

**Figure 3:**
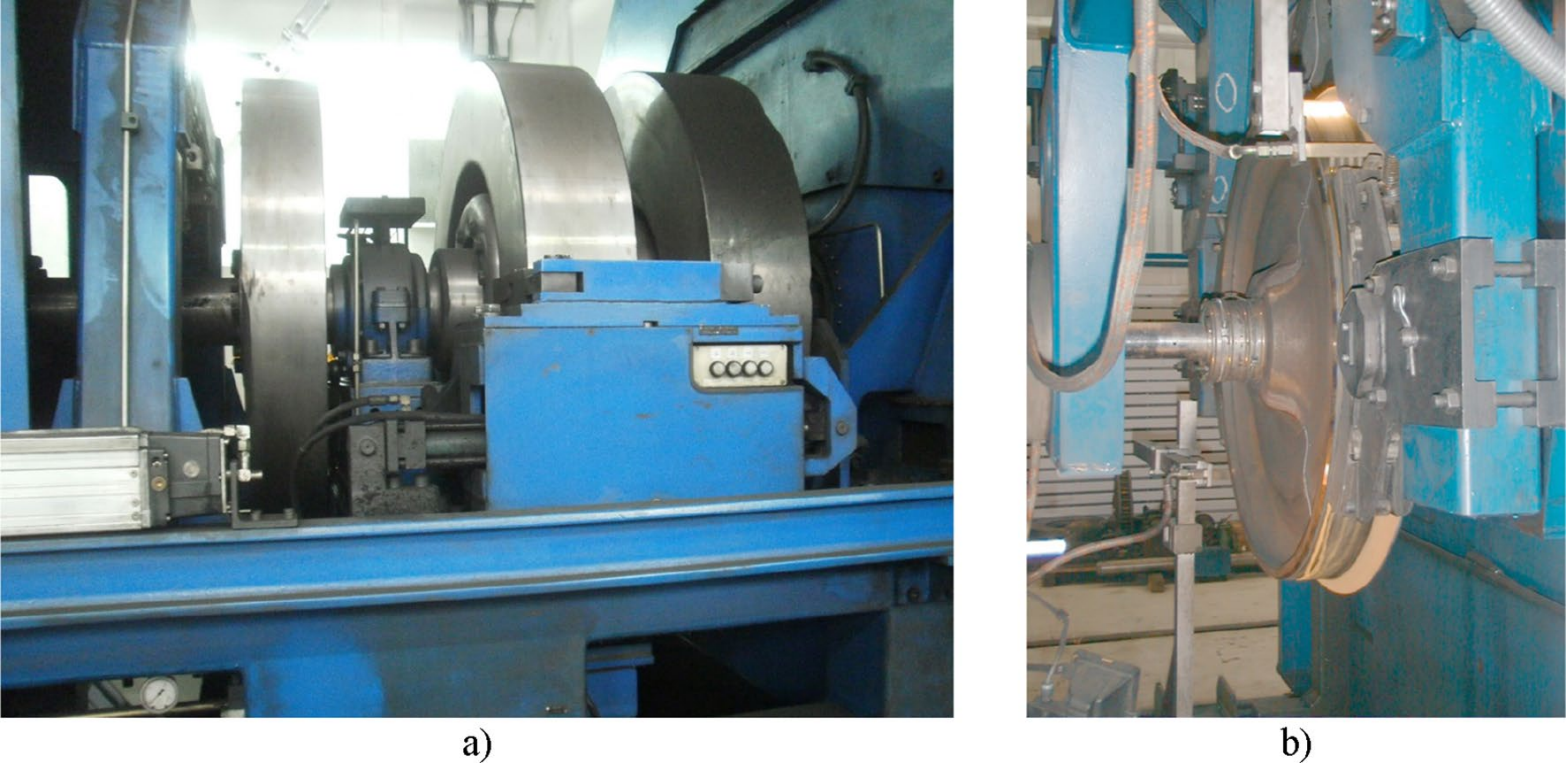
A full-scale inertia dynamometer for testing friction pairs of railway brakes at the Railway Research Institute in Warsaw: (**a**) flywheels, (**b**) test object (tread brake in 2xBgu configuration). Photographs by courtesy of the Railway Research Institute in Warsaw, Poland.

The bench test on a full-scale inertia dynamometer was carried out at the Railway Research Institute (Instytut Kolejnictwa) in Warsaw (Fig. 3). A detailed description of the stand and research methodology are presented in the article^27^.

**Figure 4:**
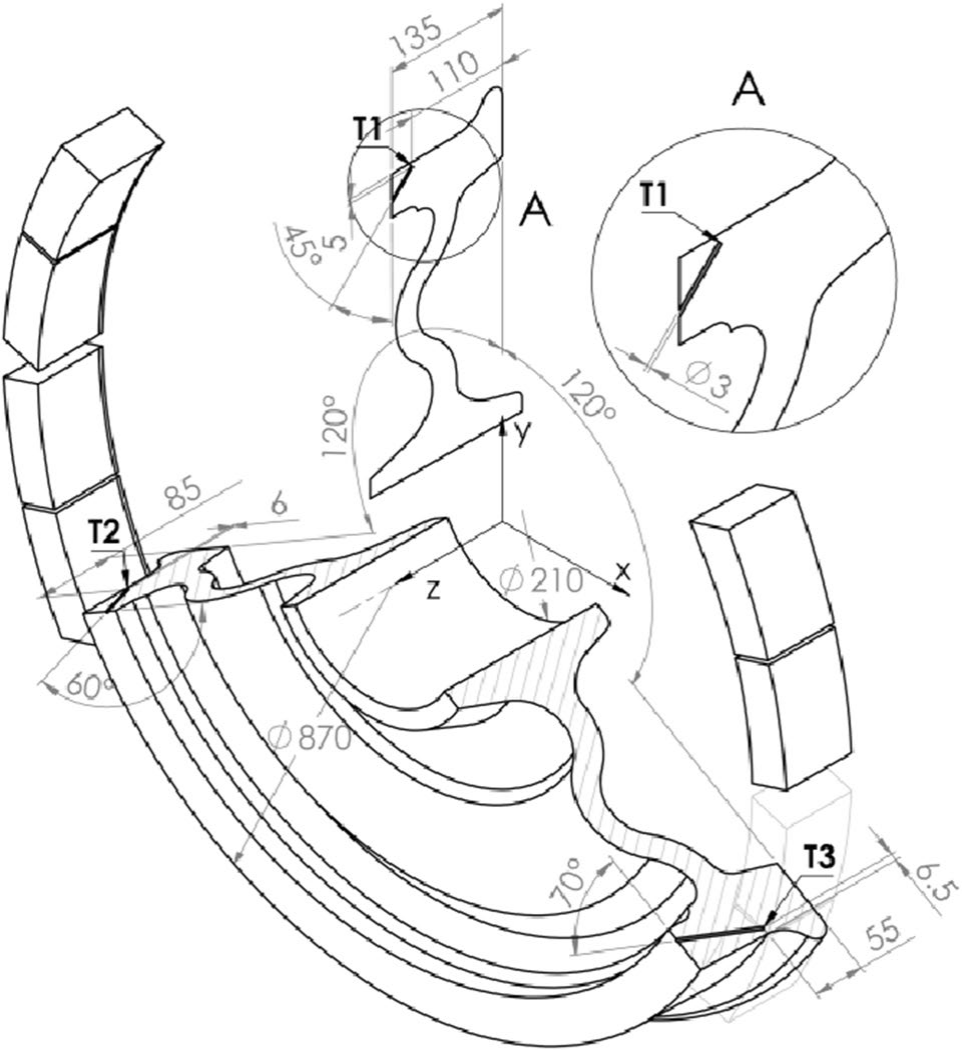
Scheme of the arrangement of thermocouples T1, T2, T3 in a railway wheel with selected dimensions.

During the test, the velocity of the wheel and the distance were measured, and the normal and tangential forces were determined, which allowed to calculate the instantaneous coefficient of friction ( $\Delta f_{a} = 0.004$—is the accuracy of determining the coefficient of friction, calculated based on errors resulting from the measurement of the braking torque and the braking force) $f_{a} = F_{t} /F_{b}$. The temperature was measured using three K-type thermocouples placed in a circle 55 mm, 85 mm and 110 mm from the outer periphery plane, 6.5 mm, 6 mm and 5 mm, respectively below the running surface, and put every 120° around the axis of rotation (Fig. 4). The temperature measurement error is $\Delta T = \pm 1.5\,^\circ {\text{C}}$ according to the manufacturer's data.

The braking sequences analyzed in this article is a section of the general test program and consist of the 12 braking-to-stops marked from H38 to H49 (Table 2). General information about the test conditions is presented in Table 3.

**Table 2:** Test program on a full-scale inertia dynamometer.

No	$V_{0}$, ${\text{km}}\,{\text{h}}^{ - 1}$	$F_{B}$, ${\text{kN}}$	$T_{0}$, ${{^\circ C}}$	$m$*,* t	Comments
H38	80	30	Ambient	7.5	Braking to a stop in dry conditions, with no cooling pause
H39			Result^a^		
H40					
H41					
H42	80	30	Ambient	7.5	Braking to a stop in dry conditions, then accelerating to a speed $V_{0}$ and 2 min of driving, then braking
H43			Result^b^		
H44					
H45					
H46	80	30	Ambient	7.5	Braking to a stop in dry conditions, then accelerating to a speed $V_{0}$ and 4 min of driving, then braking
H47			Result^b^		
H48					
H49					

^a^The initial temperature is equal to the temperature that is reached after the end of the preceding braking and the direct acceleration of the wheel to $V_{0}$.

^b^The initial temperature is equal to the temperature that is reached after the end of cooling phase (after the preceding braking and the direct acceleration of the wheel to $V_{0}$).

**Table 3:** Test conditions on a full-scale inertia dynamometer.

Parameter	Value
Brake configuration	2xBgu
Nominal dimensions of the brake block (length × width × thickness)	250 mmx 80 mm × 60 mm
Number of brake blocks	4
Wheel material	ER7 steel
Nominal diameter of the wheel	870 mm

In order to properly match the elements of the friction pair, the brake blocks were machined to obtain the shape of the friction surface adapted to the radius and profile of the wheel. Then, before the target dynamometer test, the bedding-in process was carried out, involving 30 braking-to-stops (with the initial speed of 120 ${\text{km}}\,{\text{h}}^{ - 1}$, clamping force exerted on the brake blocks of 30 kN, mass to be braked 7.5 t, and initial wheel temperature in the range 50–60 °C, except for the first brake application, where the initial temperature was equal to the ambient temperature $T_{a} = 10\,^\circ {\text{C}}$). The wheel temperature was determined as the average value measured with 3 thermocouples T1–T3 placed under the wheel tread (Fig. 4).

The data obtained from the tests are presented in the next part of the article, and compared to the values calculated on the basis of the developed numerical FE models.

## Formulating assumptions for 2D axisymmetric and 3D FE numerical models of frictional heating

The computing power of modern computers and the available CAD/CAE (computer-aided design/computer-aided engineering) software allow use of numerical calculation methods to model operating conditions of the friction interface similar to real ones, without the need to drastically simplify the geometry of analyzed objects.

As a part of this study, simulations of changes in the transient temperature fields were carried out using the proposed FE models of frictional heating for an accurate, from a geometric point of view, three-dimensional (3D) representation of the friction pair elements of the tread brake in the 2xBgu configuration (Fig. 2), and for a simplified (2D axisymmetric) model. One of the objectives of the research was to check the effect of reducing the spatial geometry of the analyzed friction pair to the 2D axisymmetric model, and the consistency of the obtained temperature fields with the experimental measurements data. To sum up, the numerical calculations of the temperature fields were carried out for two geometrical systems of the friction pair, i.e. 3D, which took into account the frictional heating of the brake blocks and the wheel; and 2D axisymmetric, where only the rotor, i.e. the railway wheel, was considered.

Geometric models, representing real objects tested on a full-size inertial test bench, were created using SolidWorks software. The position of the brake blocks in relation to the wheel tread was in accordance with the conditions of the experimental tests carried out—the contact area of the wheel-brake blocks was defined for $51\,{\text{mm}} \le z \le 131\,{\text{mm}}$, which was important for comparing the calculated and measured temperature values at each of the measuring points T1, T2 and T3 (Fig. 4). The calculation of the transient temperature fields in the wheel and brake blocks was performed using COMSOL Multiphysics^®^ software, including the Heat Transfer Module and mathematical modelling tools.

In the 3D model, the rotation of the wheel was simulated using the settings of the Translational Motion option. The components of the wheel velocity vector were as follows:

1 $$v_{x} = - yv(t)R_{w}^{ - 1} = - y\omega (t),$$

2 $$v_{y} = xv(t)R_{w}^{ - 1} = x\omega (t).$$

## Construction and verification of a finite element mesh

Table 4 presents the basic information on the division of the 2D axisymmetric and 3D spatial finite element meshes that were finally accepted for the calculations. Prior to FEM calculations, the influence of the total number of finite elements on the convergence of the obtained temperature changes at the points corresponding to the position of the thermocouples were checked. In the case of the 2D (axisymmetric) model, calculations were performed for 340, 1855 and 4540 quadratic finite elements of the second order (quadratic Lagrange). Development of the finite element model considered decisions on the total number of elements as well as its density in radial direction, in particular in the vicinity of contact between the wheel and brake blocks. The COMSOL software includes tools for the automatic creation of thin layers of finite elements with a dense mesh. The other way is to extrude 2D mesh in specific direction with applied distribution in the entire domain or object. Increasing the total number of finite elements and changing density of elements in the contact area did not improve accuracy but considerably increased the time of computation.

**Table 4:** Parameters of geometric models and applied finite elements.

	2D axisymmetric model	3D model	
	Wheel	Wheel	Brake block
Geometric shape and number of finite elements	340 square elements	83,785 tetrahedral elements and 252 pyramidal elements	1260 hexagonal elements and 10 prismatic elements
The order of finite elements	Quadratic	Quadratic	
The number of degrees of freedom	1563	147,077	

It should be added that significantly different results were obtained for a mesh made of first-order elements. To approach the results of a mesh with a standard density of second-order elements required a many times greater number of first order elements. This was especially evident in the 3D spatial model.

**Figure 5:**
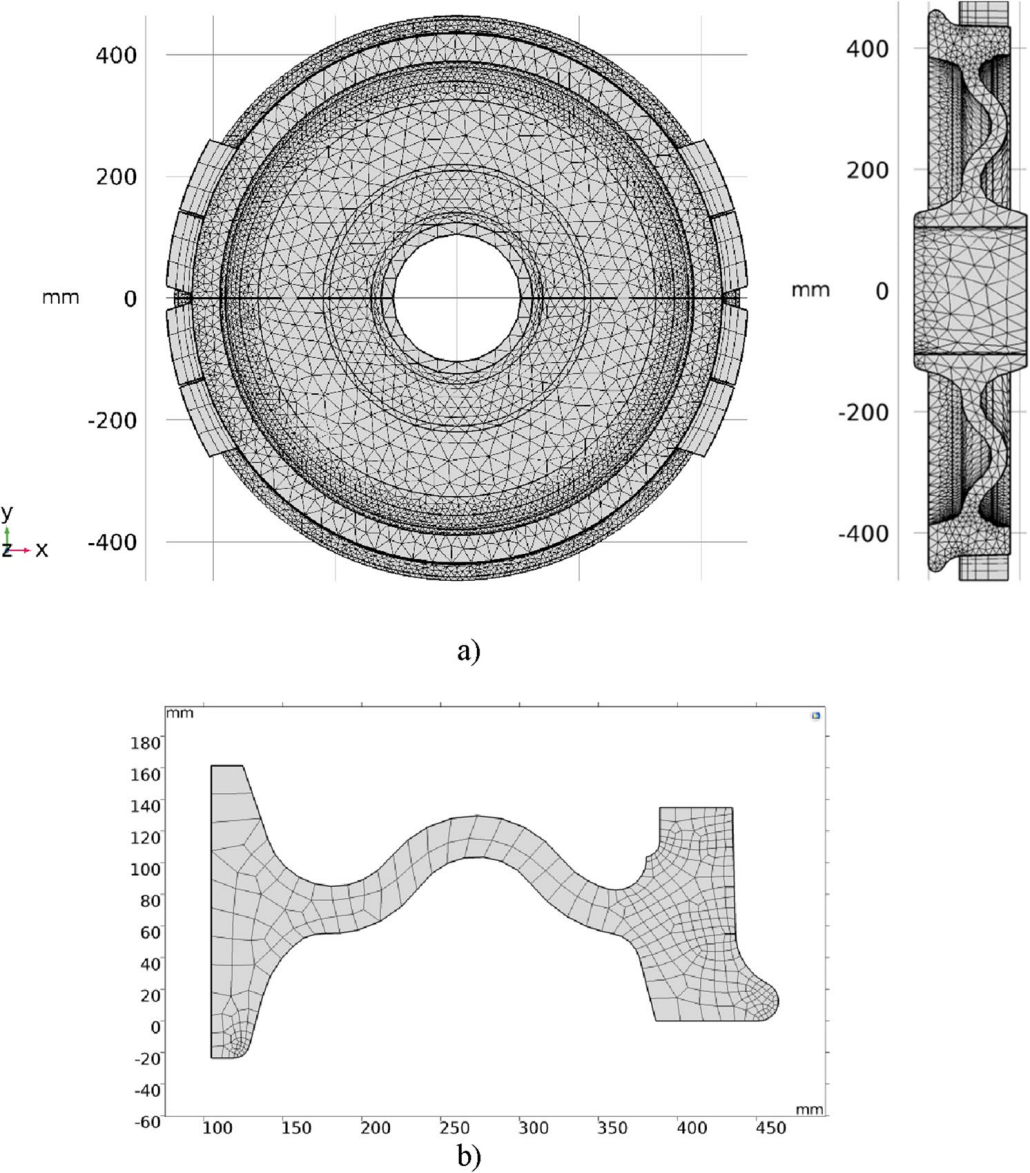
Second order (quadratic Lagrange) finite element meshes for simulation of frictional heat generation during braking: (**a**) 3D; (**b**) 2D axisymmetric.

Finally, the second-order elements (quadratic Lagrange) were used to calculate the temperature. The finite element meshes used in the calculations are presented in Fig. 5. The temperature values under the wheel running surface (in the places that correspond to the placement of thermocouples during the experimental test), determined using the finite element meshes, differed from the results obtained for the finally adopted meshes by less than 2%.

During the preparation of numerical models, the points corresponding with position of the thermocouples placed in the wheel during the test on a full-scale inertial test bench, were established, i.e., 110 mm (T1), 85 mm (T2), 55 mm (T3) from the outer periphery plane, and 5 mm, 6 mm and 6.5 mm, respectively, below the working surface, and spaced every 120° about the axis of rotation of the wheel (Fig. 4). To account for rotational movement of the thermocouples during the test, the 3D model points for which the calculated temperature values were collected also changed their position. The General Extrusion tool was used for this purpose^28^. This function allowed to determine the time-varying coordinates of points at which, after performing the calculations, the values of physical quantities were read, i.e. the temperature values. A different approach was applied for the 2D axisymmetric model. Since the axisymmetric model represented the heat flow resulting from braking in the cross-section of the wheel only, all points for further comparison with the measurement results, were placed on a common plane, while maintaining the consistency of their position in relation to the running surface and the side edge of the wheel with regard to the location of the thermocouples in the experimental test (Table 5). The division of the wheel cross-section into finite elements was made in such a way that the nodes were located at the points where data were collected for comparison with the results of experimental tests (Fig. 5b).

**Table 5:** The position of the temperature reading points under the wheel tread.

2D axisymmetric model			3D model		
No	*r*	*z*	*x*	*y*	*z*
T1	430.62	110	0	430.62	110
T2	430.24	85	– 372.6	– 215.11	85
T3	430.48	55	372.81	– 215.24	55

## Boundary conditions on the contact surface of the brake blocks and the wheel

The specific friction power, generated on the contact surfaces of the brake blocks and the wheel during braking was determined based on the experimental data carried out on a full-scale inertia dynamometer and calculated in accordance with the formula:

3 $$q(t) = p(t)V(t)f(t),$$

where: $p(t) = F_{b} (t)A^{ - 1}$, $f(t) = f_{a} (t)$, and $V(t)$ is the velocity of the vehicle.

**Figure 6:**
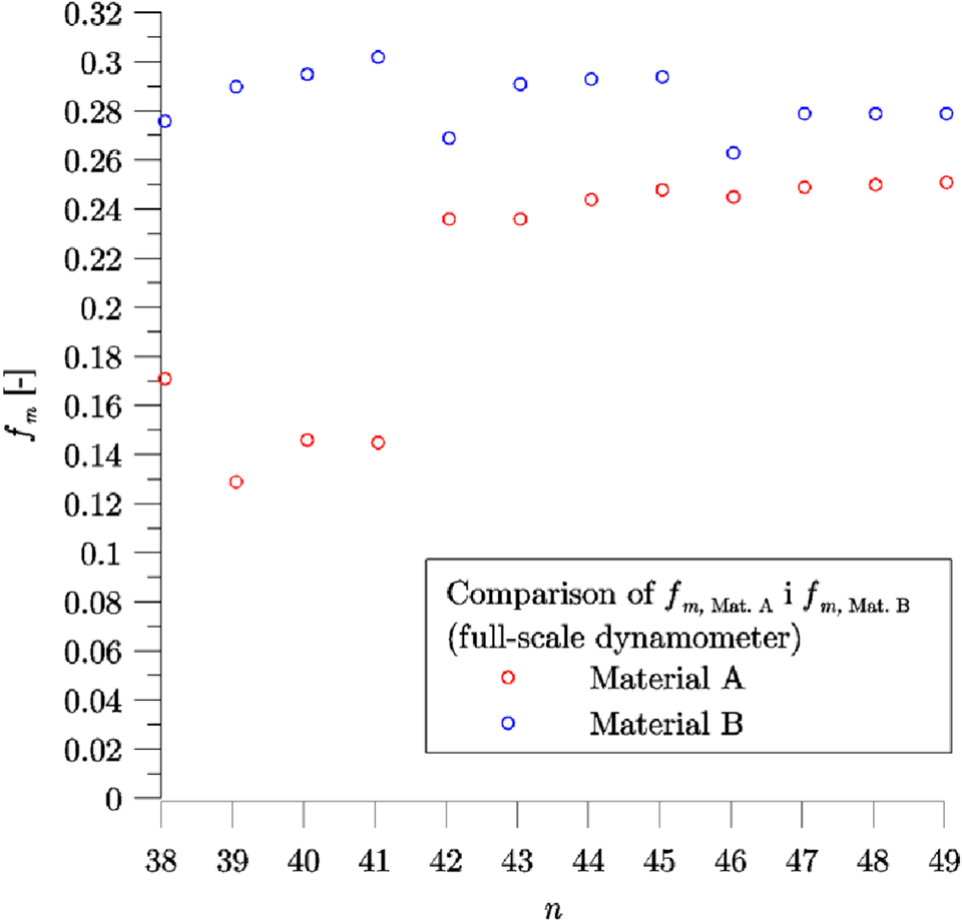
Changes of the mean coefficient of friction during the test process on a full-scale inertia dynamometer with the A and B materials of brake blocks.

**Figure 7:**
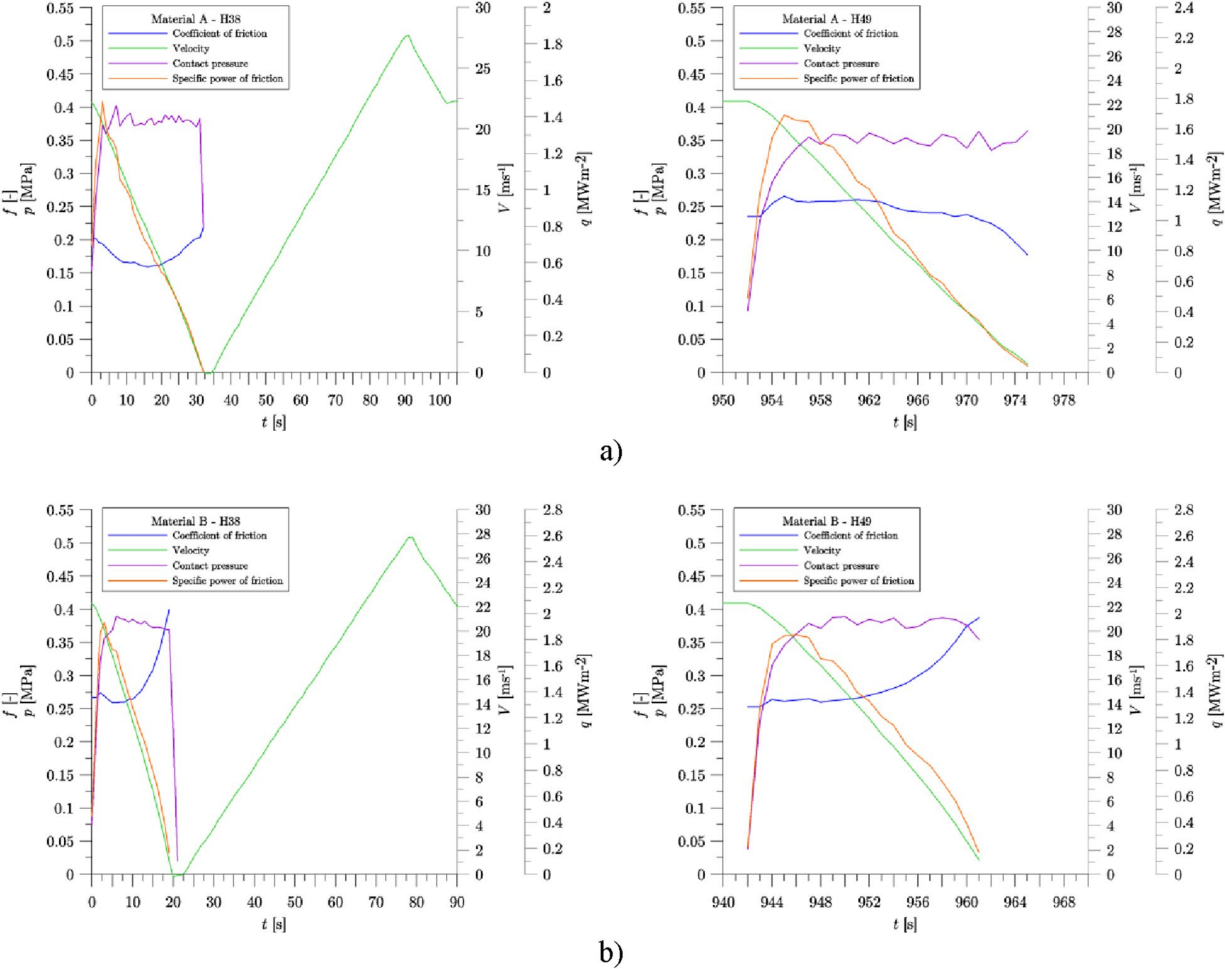
Operating parameters for H38 and H49 brake applications: (**a**) Material A; (**b**) Material B.

During the experiment, for each braking denoted H38–H49, the initial velocity of the vehicle was equal to 80 km/h, braked mass was equal to 7.5 t and the contact force was 30 kN. The changes of the mean friction coefficient (instantaneous coefficient of friction integrated over braking distance) measured during the experiment are given in Fig. 6. Sample input parameters for the first and last braking (H38 and H49) are shown in Fig. 7.

Friction materials tested in the study are characterized by a significant difference in their formulation which led to difference in their mechanical, thermophysical and frictional properties. In general coefficient of friction varies with operating conditions such as sliding speed, contact pressure and temperature, although the characteristics are specific to the friction material. The curves of the coefficient of friction shown in Fig. 7 indicate that Material B has quite strong tendency for the increase in the coefficient of friction with decreasing sliding speed (which could be attributed to metal fibers in its formulation), while Material A exhibits more stable properties in this regard or even decrease in the value of the coefficient of friction with decrease in sliding speed. Coefficient of friction in particular brake application may be also dependent on the preceding sequence of brake applications, caused by friction material being subject to changes in its structure and chemical composition related to wear and tribochemical processes on the friction interface. It is not part of the present study, but preceding brake applications (H35–H37) were characterized by relatively demanding operating conditions (initial velocity of 120 ${\text{km}}\,{\text{h}}^{ - 1}$, 160 ${\text{km}}\,{\text{h}}^{ - 1}$ and 200 ${\text{km}}\,{\text{h}}^{ - 1}$, respectively) which resulted in degraded frictional performance of Material A in subsequent brakings. Only after the sequence of 4 brake applications (H38–H41) was completed, the frictional performance of Material A recovered—the values of the coefficient of friction determined for brake applications H42–H49 are relatively stable and significantly higher as compared to values measured for brake applications H38–H41 (see Fig. 6).

To determine the value of the specific friction power *q* generated on the contact surface and the amount penetrating into the brake blocks $q_{s}$ and the wheel $q_{w}$:

4 $$q_{s} + q_{w} = q,$$

5 $$q_{s} = (1 - \gamma )q,$$

6 $$q_{w} = \gamma q.$$

Charron's formula was used^29^:

7 $$\gamma = \frac{\varepsilon }{1 + \varepsilon },$$

8 $$\varepsilon = \frac{{\lambda_{w} }}{{\lambda_{s} }}\sqrt {\frac{{K_{w} }}{{K_{s} }}} .$$

In calculations, a constant value of the heat transfer coefficient was assumed, equal for all areas of the friction pair elements $h = 65.3\,{\text{W}}\;{\text{m}}^{ - 2} \;{\text{K}}^{ - 1}$^20^. Due to the specificity of the 2D axisymmetric without brake blocks and 3D with brake blocks systems used (Fig. 5a,b), the total surface area on which heat exchange occurred with the surrounding environment, differed. In the case of the 2D axisymmetric model, on the edge representing the contact interface between the wheel and brake blocks, only heating condition (5) was applied, whereas in the 3D model in the contact area, a heating took place, and outside this area there was additional cooling.

The ambient temperature applied in the calculations was $T_{a} = 10\,^\circ {\text{C}}$. Heat dissipation by thermal radiation was not taken into account in the numerical models. Table 6 presents the values of the coefficient of thermal conductivity $\lambda_{w}$, and specific heat $c_{w}$ of the wheel material made of ER7 steel^30^. The thermophysical properties of the composite materials from which the brake blocks were made are summarized in Table 7.

**Table 6:** Thermophysical properties of the wheel material made of ER7 steel^30^.

Temperature	$\lambda_{w}$, ${\text{W}}\,{\text{m}}^{ - 1} \,{\text{K}}^{ - 1}$	$c_{w}$, ${\text{J}}\,{\text{kg}}^{ - 1} \,{\text{K}}^{ - 1}$	$\rho_{w}$, ${\text{kg}}\,{\text{m}}^{ - 3}$	$K_{w}$, ${\text{m}}^{2} {\text{s}}^{ - 1}$
0	47.3	440	7850	$1.369 \times 10^{ - 5}$
200	44.1	510	7850	$1.102 \times 10^{ - 5}$
400	39.3	570	7850	$8.783 \times 10^{ - 6}$
600	32.9	630	7850	$6.653 \times 10^{ - 6}$

**Table 7:** Thermal properties of composite materials of brake blocks.

Properties	Material A	Material B
$K_{s}$, ${\text{m}}^{2} \,{\text{s}}^{ - 1}$	$7.013 \times 10^{ - 7}$	$8.594 \times 10^{ - 7}$
$\rho_{s}$, ${\text{kg}}\,{\text{m}}^{ - 3}$	1930	2350
$c_{s}$ at 30 °C, ${\text{J}}\,{\text{kg}}^{ - 1} \,{\text{K}}^{ - 1}$	870	730
$c_{s}$ at 100 °C, ${\text{J}}\,{\text{kg}}^{ - 1} \,{\text{K}}^{ - 1}$	1040	860
$\lambda_{s}$ at 30 °C, ${\text{W}}\,{\text{m}}^{ - 1} \,{\text{K}}^{ - 1}$	1.18	1.47
$\lambda_{s}$ at 100 °C, ${\text{W}}\,{\text{m}}^{ - 1} \,{\text{K}}^{ - 1}$	1.41	1.74

To sum up the information presented above, concerning the methods of mapping the geometric shape of the friction pair, and the assumed boundary conditions, two numerical FE models used in the calculations can be distinguished:

- axisymmetric model marked 2D axisymmetric, in which imperfect thermal contact of the brake block and wheel was assumed, where the heat partition ratio was determined on the basis of the Charron formula (7), and the density of the frictional heat flux penetrating the wheel was determined taking into account the degree of cover equal to 0.364 (ratio of the wheel-brake blocks contact area to the area of the part of the wheel tread where contact with the brake blocks occurs);
- a three-dimensional model marked 3D, in which an imperfect thermal contact of the brake blocks and the wheel was assumed, and the heat partition ratio was determined based on the Charron formula (7); while, unlike in the 2D axisymmetric model, its value was determined at each time point of the calculations, and at each point of the wheel-brake blocks contact area, with the change of thermophysical properties of friction elements as a function of average temperature taken into account (the average temperature was the arithmetic mean of the wheel and brake blocks temperature at a given point in the contact area for which the $\gamma$ value was determined).

## Analysis of experimental results and numerical calculations of temperature

Repetitive braking H38–H49 (Table 2) in the experimental test and the corresponding numerical calculations were carried out for the same operating conditions in terms of the initial speed ($V_{0} = 80\,{\text{km}}\,{\text{h}}^{ - 1}$), the clamping force exerted on the brake blocks ($F_{B} = 30\,{\text{kN}}$) and the weight of the railway vehicle per wheel ($M = 7.5\,{\text{t}}$) (Fig. 6).

The difference between the H38–H41, H42–H45 and H46–H49 braking sequences lay in the duration of the cooling time of the friction pair elements (between the end of a braking and start of another braking sequence after re-accelerating the vehicle, i.e. for the braking sequence H38–H41, the successive braking occurred immediately after accelerating the wheel to the rotational speed corresponding to $V_{0}$; for H42–H45—2 min; for H46–H49—4 min after accelerating the wheel to the rotational speed corresponding to $V_{0}$).

For the first of the considered braking sequences (H38–H41), the calculated temperature values at points corresponding to the position of respective thermocouples were compared with the experimental data measured under the wheel tread. The temperature measurement results were shown taking into account the measurement error for individual thermocouples, which is $\Delta T = \pm 1.5\,^{ \circ } {\text{C}}$ according to their manufacturer's data.

Further comparison of the calculation results and experimental data concerned the average temperature values under the wheel tread, determined on the basis of the individual temperature value measured and calculated at points corresponding to the position of the thermocouples in the experimental test:

9 $$\overline{T} = \frac{{T_{1} + T_{2} + T_{3} }}{3}.$$

The maximum uncertainty of the average temperature measurement was defined as $\Delta \overline{T} = \pm 1.5\,^\circ {\text{C}}$ using the formula:

10 $$\Delta \overline{T} = \sum\limits_{i = 1}^{n} \,\left| {\frac{{\partial \overline{T} }}{{\partial T_{i} }}\Delta T_{i} } \right|.$$

where $i$ denotes a number of a thermocouple.

### Analysis of the experimental results and numerical calculations of the temperature under the wheel tread

**Figure 8:**
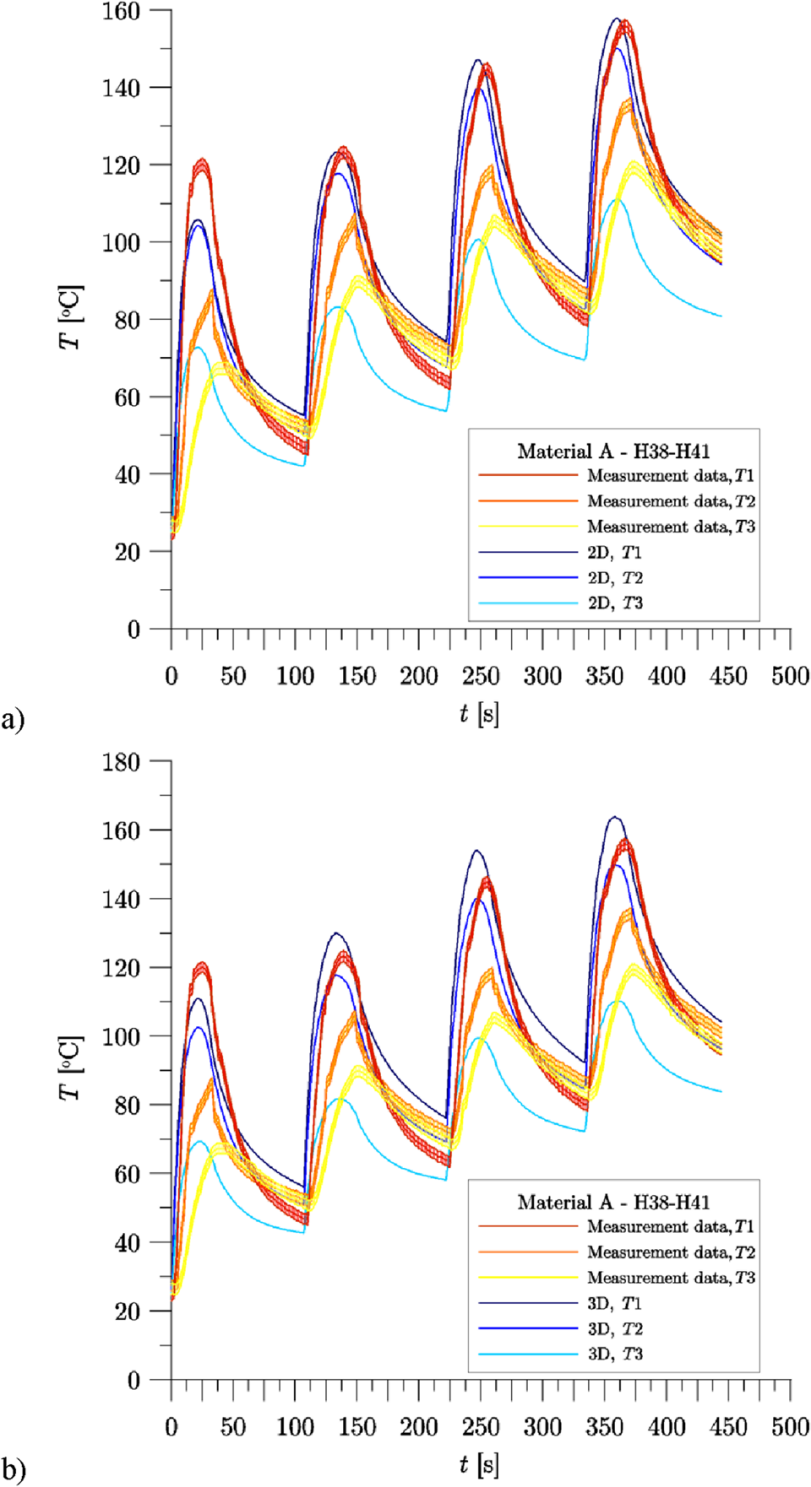
Changes of temperature under the wheel tread during braking H38–H41—comparison of experimental data and numerical calculation results for each temperature measurement points (Material A): (**a**) 2D axisymmetric model; (**b**) 3D model. Experimental data include the maximum uncertainty of the average temperature measurement (10).

In Fig. 8, for the first 4 braking applications (H38–H41) of the friction pair with Material A, the experimentally measured temperature curves from thermocouples T1–T3 and the temperature changes at corresponding points T1–T3 calculated with 2D axisymmetric and 3D models are shown.

The results of the measurements and calculations are generally in good agreement. In the case of brake blocks made of Material A, the calculated values of maximum temperature T1 during each brake applications are slightly higher than the values measured (except for the H38 and H39 brake applications for the 2D axisymmetric model, and the entire braking sequence for the 3D model), while the values of maximum temperature T3 are, in turn, slightly lower than the experimental data (except for the H38 braking for the 2D axisymmetric model). The biggest difference between calculations and measurements concerns the values of maximum temperature T2—the calculation results for each brake applications are higher than the experimental data. The best agreement among the presented results was obtained with the use of a 3D model (Fig. 8b).

**Figure 9:**
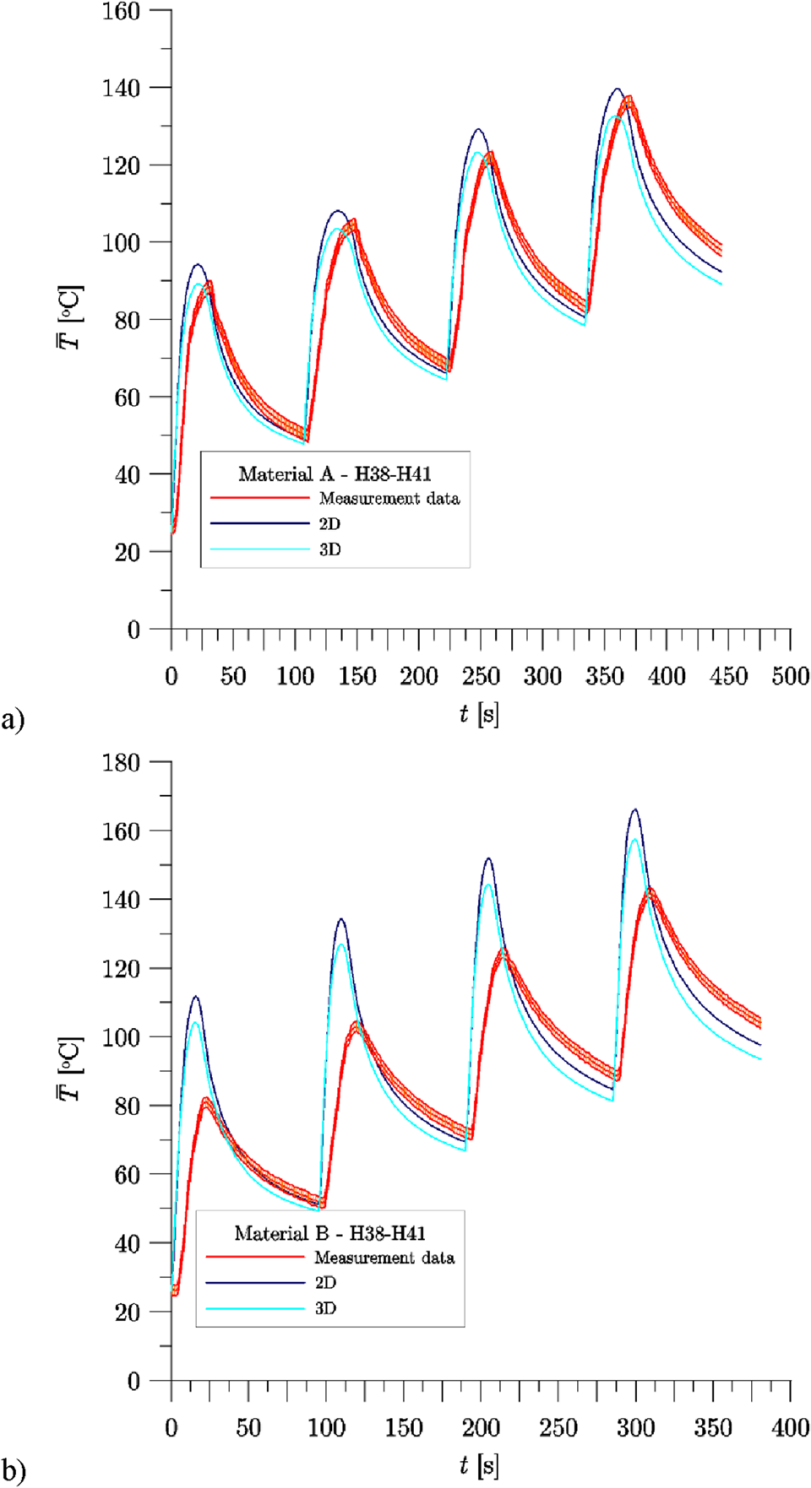
Change of average temperature under the wheel tread during braking H38–H41—comparison of experimental data and numerical calculation results for each temperature measurement points: (**a**) Material A; (**b**) Material B. Experimental data include the maximum uncertainty of the average temperature measurement (10).

Figure 9 shows the change of average temperature under the wheel tread, determined from the experimental data and numerically calculated using FE models, for the braking sequence H38–H41, for Material A (Fig. 9a) and Material B (Fig. 9b). The results of numerical calculations and experimental data of the initial temperature for all H38–H49 brake applications under the wheel tread, and the maximum average temperature during the braking of the wheel for the brake blocks made of Material A are presented in Table 8, and for Material B in Table 9.

**Table 8:** Summary of the results of numerical calculations and experimental data: the initial braking temperature and the maximum average temperature during braking under the wheel tread (Material A).

No	Initial braking temperature under the wheel tread			Maximum average temperature during braking under the wheel tread		
	Measurement results	Calculation results		Measurement results	Calculation results	
		2D axisymmetric	3D		2D axisymmetric	3D
	$\mathop {\overline{T} }\nolimits_{0,Exp.}$ (°C)	$\mathop {\overline{T} }\nolimits_{0,Num.}$ (°C)	$\mathop {\overline{T} }\nolimits_{0,Num.}$ (°C)	$\mathop {\overline{T} }\nolimits_{{{\text{max}},Exp.}}$ (°C)	$\mathop {\overline{T} }\nolimits_{{{\text{max}},Num.}}$ (°C)	$\mathop {\overline{T} }\nolimits_{{{\text{max}},Num.}}$ (°C)
H38	26.0	25.0	25.0	88.5	94.2	89.3
H39	49.8	49.2	47.9	104.7	108.1	103.5
H40	68.0	66.1	64.4	122.1	129.3	123.3
H41	83.2	80.6	78.4	136.4	139.8	132.6
H42	26.0	25.0	25.0	90.7	108.7	102.5
H43	42.0	43.2	42.0	106.9	124.3	117.4
H44	55.2	55.8	54.1	121.4	136.1	130.3
H45	65.4	65.5	63.6	133.5	142.7	137.9
H46	26.0	25.0	25.0	97.5	107.8	101.3
H47	37.3	38.0	36.9	108.7	113.2	106.4
H48	45.4	45.2	43.4	116.9	120.7	115.4
H49	51.9	50.6	48.9	123.4	123.8	118.8

**Table 9:** Summary of the results of numerical calculations and experimental data: the initial braking temperature and the maximum average temperature during braking under the wheel tread (Material B).

No	Initial braking temperature under the wheel tread			Maximum average temperature during braking under the wheel tread		
	Measurement results	Calculation results		Measurement results	Calculation results	
		2D axisymmetric	3D		2D axisymmetric	3D
	$\mathop {\overline{T} }\nolimits_{0,Exp.}$ (°C)	$\mathop {\overline{T} }\nolimits_{0,Num.}$ (°C)	$\mathop {\overline{T} }\nolimits_{0,Num.}$ (°C)	$\mathop {\overline{T} }\nolimits_{{{\text{max}},Exp.}}$ (°C)	$\mathop {\overline{T} }\nolimits_{{{\text{max}},Num.}}$ (°C)	$\mathop {\overline{T} }\nolimits_{{{\text{max}},Num.}}$ (°C)
H38	26.2	26.0	26.0	81.1	111.8	104.1
H39	51.6	51.4	49.3	103.0	134.3	127.0
H40	71.6	69.4	66.8	124.3	152.0	144.3
H41	88.7	84.7	81.3	141.9	166.1	157.5
H42	26.0	26.0	26.0	82.8	108.7	103.2
H43	42.0	43.1	42.0	99.4	128.0	122.2
H44	54.4	55.5	53.9	110.7	139.8	133.2
H45	64.6	65.4	63.4	120.6	147.4	139.7
H46	26.0	26.0	26.0	83.2	112.5	103.1
H47	36.9	39.4	37.6	92.3	121.6	116.0
H48	44.6	47.1	45.3	98.5	131.3	126.4
H49	50.3	53.3	51.5	105.6	135.8	130.2

A comparison of the measured and calculated values of the initial temperature under the wheel tread (Table 8, Fig. 9a) for Material A, shows their very good agreement—differences ($\mathop {\overline{T} }\nolimits_{0,Num.} - \mathop {\overline{T} }\nolimits_{0,Exp.}$) are negligible or insignificant for both numerical models which are discussed:

1. 2D axisymmetric: the biggest difference: 2.6 °C for H41 braking;
2. 3D: the biggest difference: 4.8 °C for H41 braking.

The convergence of the numerical calculation results with the values measured in the range of the maximum value of the average temperature under the wheel tread is good for each of the models used, and the results closer to the measurements were obtained for the calculations carried out with the use of the 3D model (Table 8). The relative difference between the calculated and measured values for the 2D axisymmetric model did not exceed 19.9%, and for the 3D model it did not exceed 13.1%.

The analysis of the temperature values contained in Table 8 shows that in the numerical calculations results, regardless of the model used (2D axisymmetric or 3D), the maximum temperature below the surface of the wheel tread is reached faster, than it results from the measurements during the test on a full-size inertial test stand: from 9 to 13 s for the 2D axisymmetric model, and from 9 to 14 s for the 3D model. The difference observed is related to the disadvantage of the temperature measurement method, that is typically used in testing brake friction elements on a full-scale inertial test stand—measurement with thermocouples mounted under the surface of the wheel tread is characterized with a significant delay.

The initial temperature values under the wheel tread, calculated for Material B, are characterized by a very good agreement with the measurement results (Table 9, Fig. 9b)—differences ($\mathop {\overline{T} }\nolimits_{0,Num.} - \mathop {\overline{T} }\nolimits_{0,Exp.}$) are negligibly small or insignificant for both proposed numerical models, although greater than in the calculations carried out for Material A:

1. 2D axisymmetric: the biggest difference: 0.2 °C for H39 braking;
2. 3D: the biggest difference: 7.4 °C for H41 braking.

For Material B, the relative difference between the calculated and measured values of the maximum average temperature under the wheel tread $\delta_{{\overline{T} ,{\text{max}}}}$ was generally greater than for Material A (Table 9, Fig. 8b), and also in this case the results for the calculations carried out with the use of the 3D model were closer to the experimental data. The relative difference between the calculated and measured values for the 2D axisymmetric model did not exceed 38%, and for the 3D model it did not exceed 28%.

1. 2D axisymmetric: from 17.0% (H41, $\Delta_{{\overline{T} ,{\text{max}}}} \approx 24.2\,^\circ$C) to 38.0% (H38, $\Delta_{{\overline{T} ,{\text{max}}}} \approx 30.8\,^\circ$C);
2. 3D: from 11.0% (H41, $$\Delta_{{\overline{T} ,{\text{max}}}} \approx 15.6\,^\circ$$C) to 28.5% (H38, $$\Delta_{{\overline{T} ,{\text{max}}}} \approx 23.1\,^\circ$$C).

As in the case of Material A, the results of the numerical calculations performed for Material B show that the maximum temperature under the wheel tread is reached faster than in the experimental data (Table 9). The difference is 6 s to 10 s for a 2D axisymmetric model, and 6 s to 10 s for a 3D model. For the H38–H42 brake applications (Fig. 8b), the difference between the experimentally determined and the calculated (using a 3D model) time to reach the maximum temperature under the wheel tread is 2 s to 5 s smaller for Material B compared to the results for Material A (i.e. in these calculations the temperature rise under the wheel tread was slightly slower than in the other cases). A similar observation applies to the results of calculations carried out for H38–H42 braking using a 2D model, although the difference is slightly smaller (from 1 to 4 s).

### Analysis of the results of numerical calculations of temperature on the working surface (the wheel tread)

Comparison of the results of numerical calculations and experimental data on the temperature distribution under the wheel tread allowed to confirm the usefulness of the proposed numerical models for estimating the temperature value during frictional heating of the friction elements of the railway tread brake. In this part of the article, the results of calculations concerning the temperature distribution on the contact surface of the brake block and the wheel will be discussed.

Table 10 summarizes the calculated values of the average initial and maximum temperature on the contact surface of wheel and brake blocks made of Material A and Material B. The average temperature on the surface is presented as the mean temperature of the contact area of the brake block and wheel for the 2D axisymmetric model, and the average of 3 points on the surface for the 3D model (the coordinates of the points are presented in Table 11). It results from the calculation method in the 3D model, where the value of the heat partition ratio $\gamma$ at each time point of the calculations was determined for the thermophysical properties of the friction elements dependent on the temperature on the contact surface, which was the arithmetic mean of the temperature of the wheel and brake blocks at a given point of the contact area. Therefore, it was necessary to indicate data reading points on the wheel tread, regardless of the defined common contact area.

**Table 10:** Results of numerical calculations: initial temperature and maximum temperature on the contact surface during braking.

No	Material A				Material B			
	Initial temperature		Maximum temperature		Initial temperature		Maximum temperature	
	2D	3D	2D	3D	2D	3D	2D	3D
	$\mathop {\overline{T} }\nolimits_{0,Num.}$ (°C)	$\mathop {\overline{T} }\nolimits_{0,Num.}$ (°C)	$\mathop {\overline{T} }\nolimits_{{{\text{max}},Num.}}$ (°C)	$\mathop {\overline{T} }\nolimits_{{{\text{max}},Num.}}$ (°C)	$\mathop {\overline{T} }\nolimits_{0,Num.}$ (°C)	$\mathop {\overline{T} }\nolimits_{0,Num.}$ (°C)	$\mathop {\overline{T} }\nolimits_{{{\text{max}},Num.}}$ (°C)	$\mathop {\overline{T} }\nolimits_{{{\text{max}},Num.}}$ (°C)
H38	25.0	25.0	121.7	105.3	26.0	26.0	151.6	128.4
H39	50.6	47.8	130.8	116.8	52.9	49.3	179.0	152.4
H40	68.0	64.2	155.3	138.6	71.6	66.7	196.5	169.7
H41	82.7	78.2	164.1	146.9	87.2	81.1	215.0	182.7
H42	25.0	25.0	148.1	125.7	26.0	26.0	147.0	126.4
H43	43.8	41.8	164.4	140.6	43.6	41.8	170.1	147.5
H44	56.5	53.9	176.6	154.1	56.1	53.7	182.3	158.0
H45	66.1	63.4	182.8	161.2	66.1	63.1	190.3	164.1
H46	25.0	25.0	148.3	124.7	26.0	26.0	151.6	126.1
H47	38.1	36.8	150.1	127.9	39.5	37.5	162.1	140.1
H48	45.3	43.3	158.9	137.7	47.2	45.1	173.1	151.2
H49	50.7	48.7	162.1	140.8	53.3	51.3	176.7	154.1

**Table 11:** Position of the temperature collection points on the contact surface for the 3D model.

No	$x$	$y$	$z$
T_4_	− 217.81	377.26	110
T_5_	− 218.12	− 377.79	85
T_6_	436.98	0	55

**Figure 10:**
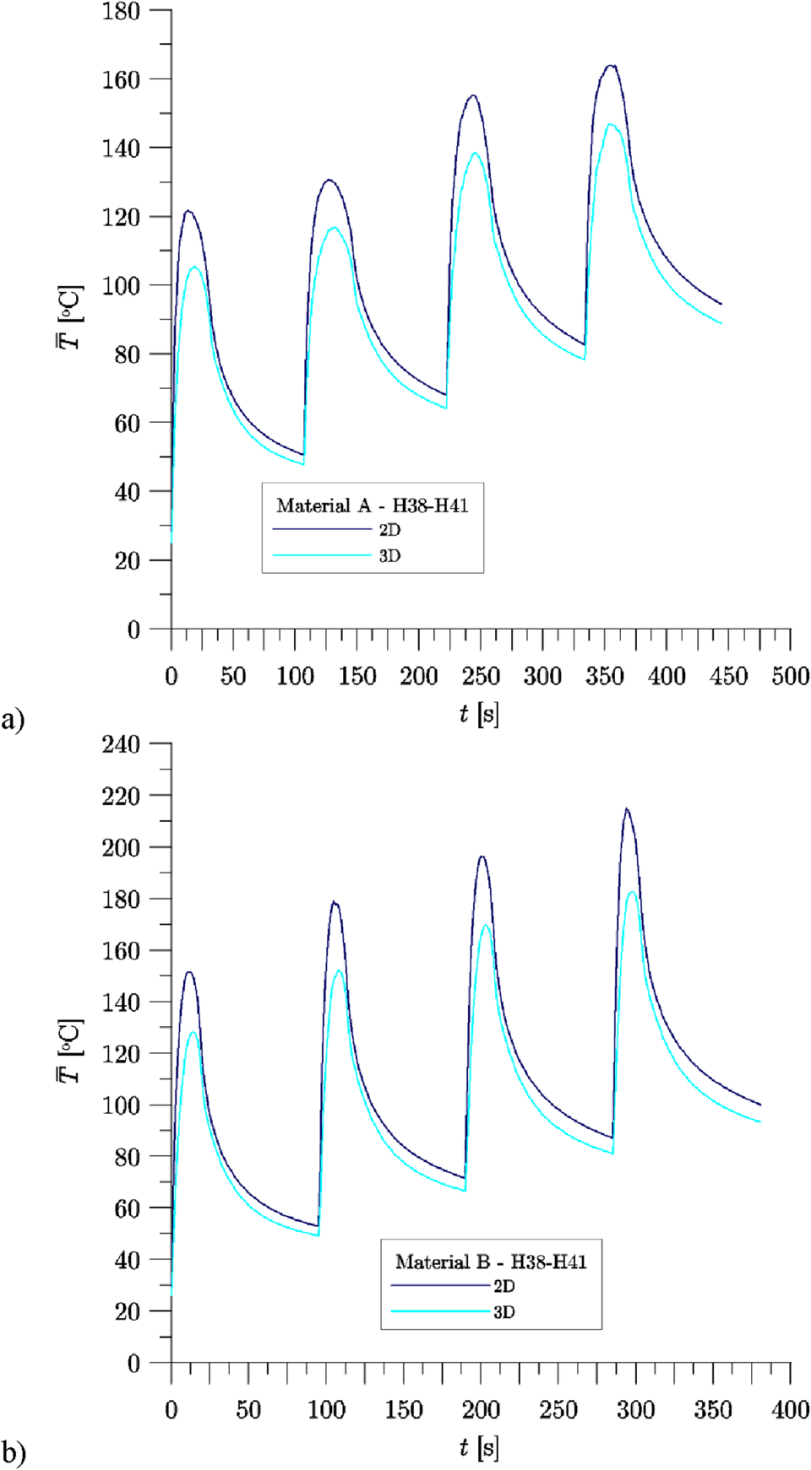
Changes of temperature on the wheel tread during braking H38–H41—results of numerical calculations for 2D axisymmetric and 3D models: (**a**) Material A, (**b**) Material B.

Figure 10 shows the comparison between changes of average temperature on the contact surface of the brake blocks and wheels, calculated numerically using 2D axisymmetric and 3D models for the H38–H41 braking sequence, for Material A (Fig. 10a) and Material B (Fig. 10b). The differences between the average surface temperature values determined in the calculations carried out with the 2D axisymmetric and 3D models were greater than in the case of the temperature determined under the contact surface (compare Figs. 9 and 10). The lowest values of the initial and average maximum temperature on that surface were obtained for respective braking in each case in the calculations with the use of the 3D model. The largest difference between the $\mathop {\overline{T} }\nolimits_{0,Num.}$ values taking into account the 2D axisymmetric and 3D models, was 8.4 °C—brake application H41, Material B. The comparison of the results of numerical calculations obtained for the 2D and 3D models shows that the differences between the values $\mathop {\overline{T} }\nolimits_{{{\text{max}},Num.}}$ were greater than in the case $\mathop {\overline{T} }\nolimits_{0,Num.}$ and exceeded even over 40 °C. Regardless of the material of the brake blocks analyzed (Material A or Material B), for each of the brake applications the obtained values $\mathop {\overline{T} }\nolimits_{{{\text{max}},Num.}}$ were higher for the 2D axisymmetric model compared to the 3D model.

The comparison of the values $\mathop {\overline{T} }\nolimits_{{{\text{max}},Num.}}$ determined in individual brake application for both analyzed materials A and B (Tables 8 and 9) shows that higher values of the average surface temperature were achieved in the simulations of braking with brake blocks made of Material B. The only exception to this regularity are the results of calculations performed with the 2D axisymmetric model for H42 brake application. It is worth drawing attention to the fact, that in each of the three analyzed brake sequences (H38–H41, H42–H45, H46–H49) the differences between the estimated values for both materials were always lower for the first braking. The greatest difference in the achieved values $\mathop {\overline{T} }\nolimits_{{{\text{max}},Num.}}$ concerns the H38–H41 braking sequences, for which there was also the greatest difference noted between the values of the coefficient of friction measured for both materials.

The differences described above result essentially from the frictional properties of the materials tested. In all braking applications, in the range that was analyzed, brake blocks made of Material B were characterized by a higher coefficient of friction compared to those made of Material A. Since the set braking operating parameters were the same ($V_{0}$ = 80 km/h, $F_{B}$ = 30 kN and $M$ = 7.5 t), differences in the friction coefficient values resulted in differences in the time profiles of the friction heat flux density during braking.

The performed calculations showed that both models reflect well the changes in the temperature of the wheel during braking. The ratios of calculation time required to determine braking sequences H38– H41, H42–H45, H46–H49 for friction couples Material A/ ER7 steel and Material B/ER7 steel versus real braking time are shown in Table 12. The simulations of braking were carried out at CPU Intel^®^ Xeon^®^ E5-2698 v4 @ 2.20 GHz and RAM 64 GB (DDR4).

**Table 12:** Braking times and computation times obtained for 2D axisymmetric and 3D models.

	Material A/ER7 steel			Material B/ER7 steel		
	Real braking time	Calculation time using 2D axisymmetric model/real braking time	Calculation time using 3D model/real braking time	Real braking time	Calculation time using 2D axisymmetric model/real braking time	Calculation time using 3D model/real braking time
H38–41	444 s	0.196	11.423	380 s	0.095	9.697
H42–45	790 s	0.327	13.347	771 s	0.079	10.545
H46–49	1268 s	0.543	17.846	1256 s	0.522	13.205

As can be seen from the presented data, by using the 2D axisymmetric model, it is possible to obtain a solution 32.9–58.3 times faster (Material A/ER7 steel) with a braking time of 1268 s and 444 s, respectively. For the Material B/ER7 steel pair this difference was greater and varied from 25.3 to 133.5 with a braking time of 1256 s and 771 s, respectively. It can be seen that the longer the braking time, the greater the gain in computing time using the 2D axisymmetric model with respect to the 3D model.

## Conclusions

The analysis of transient temperature changes of the wheel obtained from numerical calculations and its comparison with the measurements carried out during experimental tests for three braking sequences H38–41, H42–H45 and H46–H49, allow to draw the following conclusions:

- The results of the performed numerical calculations are generally well compliant with the experimental data. While for one of the materials (Material A) this agreement is better compared to the results for the other material (Material B). Therefore the assumptions made and the data characterizing the friction pair introduced into the numerical models were not sufficient to fully reproduce the frictional heating process.
- The performed computer simulations show differences in the average temperature changes under the wheel tread during braking, between the 2D axisymmetric and 3D models for a specific friction material. When analyzing the adopted boundary conditions, it should be stated that this is an effect of convection cooling, different in both models because of the size of the free surface area of the wheel rubbing path. In the 2D axisymmetric model unlike the 3D, the cooling did not take into account the presence of brake blocks. The second condition, different in the developed models, concerned the heat distribution. In the 2D axisymmetric model, the heat partition coefficient was constant, while in the 3D model, the HPC took into account changes in thermophysical properties under the influence of the surface temperature of the contacting elements. Due to the greatest change of $\gamma$ did not exceed 2%, it was found that the heat distribution condition did not significantly affected the wheel temperature, taking into account the 2D axisymmetric and 3D models.
- Analyzing the average temperature evolutions of the wheel presented in Figs. 9 and 10 (H38–H41), it can be observed that at the starting points of subsequent braking applications, the calculated values (2D axisymmetric and 3D models, Material A, Material B) almost coincided with the experimental data or the slight temperature difference was maintained during entire sequence. This means that the heat balance, i.e. the amount of heat dissipated through convection to the surrounding environment and the heat generated during friction, were equalized.
- The consistency of the temperature changes calculated and measured (H38–H41), was much better, if the mean temperature values from the three thermocouples were compared. When collating the individual thermocouples, it was difficult to find the coincidence between the calculated and measured values.
- In order to improve the overall agreement of the numerical calculations with experimental tests, other methods of determining the heat separation should be considered, e.g. perfect thermal contact condition or taking into account thermal contact resistance.

## Data Availability

The datasets used and/or analysed during the current study available from the corresponding author (m.kuciej@pb.edu.pl) on a reasonable request.

## List of symbols

$A$: Nominal area of the contact region between brake shoe and wheel (${\text{m}}^{2\,}$)
$c$: Specific heat capacity (${\text{J}}\,{\text{kg}}^{ - 1\,} {\text{K}}^{ - 1}$)
$f$: Coefficient of friction (dimensionless)
$f_{a}$: Instantaneous coefficient of friction (dimensionless)
$f_{m}$: Mean coefficient of friction (calculated per UIC 541-4)
$F_{B}$: Total nominal braking force exerted on the friction elements (${\text{N}}$)
$F_{b}$: Total instantaneous braking force exerted on the friction elements (${\text{N}}$)
$F_{t}$: Instantaneous braking force (${\text{N}}$)
$h$: Heat transfer coefficient (${\text{W}}\,{\text{m}}^{ - 2\,} {\text{K}}^{ - 1}$)
$$K$$: Thermal diffusivity ($${\text{m}}^{2\,} {\text{s}}^{ - 1}$$)
$m$: Braking mass per one wheel (${\text{kg}}$)
$$M$$: Braking torque ($${\text{N}}\,{\text{m}}$$)
$$p$$: Contact pressure ($${\text{MPa}}$$)
$q$: Specific friction power (${\text{W}}\,{\text{m}}^{ - 2\,}$)
$$r$$: Radial coordinate ($${\text{m}}$$)
$r_{eq}$: Equivalent radius of the contact region (${\text{m}}$)
$R_{w}$: Radius of the wheel (${\text{m}}$)
$$t$$: Time ($${\text{s}}$$)
$$T$$: Temperature (°C)
$$T_{a}$$: Ambient temperature (°C)
$$T_{0}$$: Initial temperature (°C)
$$\overline{T }$$: Average temperature(°C)
T1–T3: Temperature at specific location inside the wheel (°C)
T4–T6: Temperature at specific location on the contact surface of the wheel (°C)
$V$: Velocity on the equivalent radius of the wheel (vehicle velocity) (${\text{m}}\,{\text{s}}^{ - 1}$)
$V_{0}$: Initial vehicle velocity (${\text{m}}\,{\text{s}}^{ - 1}$)
$$x,y,z$$: Axial coordinates ($${\text{m}}$$)

## Greek symbols

$\Delta f_{a}$: Accuracy of determining the coefficient of friction, calculated based on errors resulting from the measurement of the braking torque and the braking force (dimensionless)
$\gamma$: Heat partition coefficient (dimensionless)
$$\lambda$$: Thermal conductivity ($${\text{W}}\,{\text{m}}^{ - 1} \,{\text{K}}^{ - 1}$$)
$$\rho$$: Mass density ($${\text{kg}}\,{\text{m}}^{ - 3}$$)
$\omega$: Angular velocity of the wheel (${\text{rad}}\,{\text{s}}^{ - 1}$)
$\omega_{0}$: Initial angular velocity of the wheel (${\text{rad}}\,{\text{s}}^{ - 1}$)

## Subscripts

$s,w$: Brake block, wheel
$$E$$: Experiment
$${\text{Num}}$$: Numerical calculation

## References

[1] Cantone L, Ottati A (2018). Modelling of friction coefficient for shoes type LL by means of polynomial fitting. Open Transp. J..

[2] Michali M, Emrouznejad A, Dehnokhalaji A, Clegg B (2021). Noise-pollution efficiency analysis of European railways: A network DEA model. Transp. Res. Part D Transp. Environ..

[3] European Commission, Position Paper on the European strategies and priorities for railway noise abatement, Brussels. (2003).

[4] Commission regulation (EU) No 1304/2014 on the technical specification for interoperability relating to the subsystem ‘rolling stock—noise’ amending Decision 2008/232/EC and repealing Decision 2011/229/EU. (2014).

[5] Yanar H, Purcek G, Demirtas M, Ayar HH (2022). Effect of hexagonal boron nitride (h-BN) addition on friction behavior of low-steel composite brake pad material for railway applications. Tribol. Int..

[6] Monreal P, Clavería I, Arteta P, Rouzaut T (2021). Effect of modified novolac resins on the physical properties and friction performance of railway brake blocks. Tribol. Int..

[7] Monreal-Pérez P, González J, Iraizoz A, Bilbao U, Clavería I (2022). Effect of the steel fiber length on the friction performance and wear mechanism of railway brake shoes. Tribol. Int..

[8] Wasilewski P (2017). Experimental study on the effect of formulation modification on the properties of organic composite railway brake shoe. Wear.

[9] Wasilewski P (2018). Full-scale dynamometer test of composite railway brake shoes—Study on the effect of the reinforcing fibre type. Acta Mech. Autom..

[10] Vernersson T (2007). Temperatures at railway tread braking. Part 1: Modelling. Proc. Inst. Mech. Eng. Part. F J. Rail. Rapid. Transit..

[11] Vernersson T (2007). Temperatures at railway tread braking. Part 2: Calibration and numerical examples. Proc. Inst. Mech. Eng. Part. F J. Rail. Rapid. Transit..

[12] Vernersson T, Lundén R (2007). Temperatures at railway tread braking. Part 3: wheel and block temperatures and the influence of rail chill. Proc. Inst. Mech. Eng. Part F J. Rail. Rapid. Transit..

[13] Wasilewski P (2020). Frictional heating in railway brakes: A review of numerical models. Arch. Comput. Methods Eng..

[14] Günay M, Korkmaz ME, Özmen R (2020). An investigation on braking systems used in railway vehicles. Eng. Sci. Technol. Int. J..

[15] Vakkalagadda MRK, Vineesh KP, Racherla V (2015). Estimation of railway wheel running temperatures using a hybrid approach. Wear.

[16] Somà A, Aimar M, Zampieri N (2021). Simulation of the thermal behavior of cast iron brake block during braking maneuvers. Appl. Sci..

[17] Walia MS, Vernersson T, Lundén R, Blennow F, Meinel M (2019). Temperatures and wear at railway tread braking: Field experiments and simulations. Wear.

[18] Teimourimanesh S, Vernersson T, Lundén R (2016). Thermal capacity of tread-braked railway wheels. Part 1: Modelling. Proc. Inst. Mech. Eng. Part F J. Rail. Rapid. Transit..

[19] Teimourimanesh S, Vernersson T, Lundén R (2016). Thermal capacity of tread-braked railway wheels. Part 2: Applications. Proc. Inst. Mech. Eng. Part F J. Rail. Rapid. Transit..

[20] Kuciej M, Grzes P, Wasilewski P (2020). A comparison of 3D and 2D FE frictional heating models for long and variable applications of railway tread brake. Materials (Basel)..

[21] Walia MS, Esmaeili A, Vernersson T, Lundén R (2018). Thermomechanical capacity of wheel treads at stop braking: A parametric study. Int. J. Fatigue.

[22] Handa K, Ikeuchi K, Morimoto F (2020). Temperature-dependent wear of tread-braked railway wheels. Wear.

[23] Faccoli M, Provezza L, Petrogalli C, Ghidini A, Mazzù A (2019). Effects of full-stops on shoe-braked railway wheel wear damage. Wear.

[24] Faccoli M, Provezza L, Petrogalli C, Ghidini A, Mazzù A (2020). A small-scale experimental study of the damage due to intermittent shoe braking on the tread of high-speed train wheels. Tribol. Trans..

[25] Mańka A, Sitarz M (2016). Effects of a thermal load on the wheel/brake-block subsystem: The thermal conicity of railway wheels. Proc. Inst. Mech. Eng. Part F J. Rail. Rapid. Transit..

[26] Teimourimanesh S, Vernersson T, Lundén R (2014). Modelling of temperatures during railway tread braking: Influence of contact conditions and rail cooling effect. Proc. Inst. Mech. Eng. Part F J. Rail. Rapid. Transit..

[27] Konowrocki R, Kukulski J, Walczak SG (2013). Dynamic interaction of cleansing brake insert for high speed train—Experimental investigation. WUT J. Transp. Eng..

[28] COMSOL Multiphysics^®^ v. 5.3. www.comsol.com. COMSOL AB.

[29] Charron, F. Partage de la chaleur entre deux corps frottants. *Publ. Sci. Tech. du Min. l’Air* **182**, (1943).

[30] Faccoli M, Ghidini A, Mazzù A (2018). Experimental and numerical investigation of the thermal effects on railway wheels for shoe-braked high-speed train applications. Metall. Mater. Trans. A.

